# Domeless receptor loss in fat body tissue reverts insulin resistance induced by a high-sugar diet in *Drosophila melanogaster*

**DOI:** 10.1038/s41598-021-82944-4

**Published:** 2021-02-05

**Authors:** Fernanda Lourido, Daniela Quenti, Daniela Salgado-Canales, Nicolás Tobar

**Affiliations:** Cellular Biology Laboratory, Institute of Nutrition and Food Technology (INTA), University of Chile, Av. El Líbano, 5524 Macul, Santiago Chile

**Keywords:** Mechanisms of disease, Insulin signalling, Lipid signalling

## Abstract

Insulin resistance is a hallmark of type 2 diabetes resulting from the confluence of several factors, including genetic susceptibility, inflammation, and diet. Under this pathophysiological condition, the dysfunction of the adipose tissue triggered by the excess caloric supply promotes the loss of sensitivity to insulin at the local and peripheral level, a process in which different signaling pathways are involved that are part of the metabolic response to the diet. Besides, the dysregulation of insulin signaling is strongly associated with inflammatory processes in which the JAK/STAT pathway plays a central role. To better understand the role of JAK/STAT signaling in the development of insulin resistance, we used a simple organism, *Drosophila melanogaster*, as a type 2 diabetes model generated by the consumption of a high-sugar diet. In this model, we studied the effects of inhibiting the expression of the JAK/STAT pathway receptor Domeless, in fat body, on adipose metabolism and glycemic control. Our results show that the Domeless receptor loss in fat body cells reverses both hyperglycemia and the increase in the expression of the insulin resistance marker Nlaz, observed in larvae fed a high sugar diet. This effect is consistent with a significant reduction in Dilp2 mRNA expression and an increase in body weight compared to wild-type flies fed high sugar diets. Additionally, the loss of Domeless reduced the accumulation of triglycerides in the fat body cells of larvae fed HSD and also significantly increased the lifespan of adult flies. Taken together, our results show that the loss of Domeless in the fat body reverses at least in part the dysmetabolism induced by a high sugar diet in a *Drosophila* type 2 diabetes model.

## Introduction

Diabetes Mellitus is the most prevalent metabolic disorder in humans and is considered the seventh leading cause of death in the world by the WHO^1^. The most frequent types of this disease are: type 1 and type 2, with type 2 diabetes (T2D) accounting for around 90% of all diabetes cases in the adult population. T2D is a heterogeneous disease characterized by deficient insulin secretion by pancreatic islet β-cells in the context of impaired insulin sensitivity, termed insulin resistance^2^.

Insulin is an endocrine peptide hormone secreted in response to increased plasma levels of glucose and amino acids^3,4^. Its function is to promote the control of metabolism at the cellular and whole-organism level, stimulating the uptake of glucose and promoting lipogenesis. Additionally, insulin suppresses hepatic gluconeogenesis and lipolysis in adipocytes^4–6^. Insulin resistance (IR) is defined when target tissues need an excessive amount of insulin to accomplish their metabolic task. If pancreatic β-cells are able to compensate for insulin deficiency by hypersecretion of the hormone, glucose tolerance remains normal^7,8^.

Furthermore, diverse diseases associated with obesity, such as T2D, cardiovascular disease, and cancer, have in common a low-grade chronic inflammatory status, which is characterized by elevated circulating pro-inflammatory cytokines^9–11^. Moreover, inflammation has been proposed as one of the connection points between obesity and IR^12^. The increase in circulating levels of cytokines, observed as a result of excess caloric intake, promotes the development of IR by modulating signal transduction pathways that appear to be evolutionarily conserved^13,14^.

The action of these pro-inflammatory cytokines produces a state that promotes adipose tissue dysfunction and the activation of the immune system characteristic of many chronic non-communicable diseases, such as diabetes^15^. Cytokines are produced in different cell types and regulate various processes through diverse target tissues. For example, tumor necrosis factor-alpha (TNF)-α, an inflammatory marker mainly produced by monocytes and macrophages, is strongly expressed by adipose tissue in animal models of obesity and T2D^16^. IL-6 is another pro-inflammatory cytokine involved in obesity-related insulin resistance, although it has a more controversial role. On the one hand, it has been observed that increased levels of Interleukin-6 (IL-6) can promote IR in obesity conditions^17^ and, on the other hand, IL-6 deficiency can exacerbate liver IR and inflammation in mice subjected to hypercaloric diets^18^.

In this sense, adipocytes are responsible for the production of different enzymes, hormones, cytokines, and growth factors that modulate various processes^19^. In the context of adipose dysfunction, it has been observed that different adipokines, such as leptin and IL-6, are capable of activating the JAK/STAT pathway. Recent evidence supports that this highly conserved pathway is responsible for maintaining homeostasis and that its deregulation promotes the development of IR and pathologies such as obesity and diabetes (reviewed in^20^). Among mammals, the JAK/STAT pathway plays a role in adipose tissue function, modulating lipid and glucose metabolism and insulin signaling (reviewed in^21^).

Besides, the *Drosophila* model has become a versatile and reliable tool for metabolic studies due to: (1) plenty of many structural and functional similarities, which allow associating metabolic processes in flies with those observed in mammals^22–24^. (2) Some mechanisms and molecular components that participate in glucose homeostasis are conserved partially between flies and humans^25^. (3) Being an easily manipulated experimental model due to the possibility of incorporating different agents in the semi-solid diet and (4) the determinant influence of metabolic factors that facilitate nutritional evaluation using recognized development and phenotypical markers.

The mammalian insulin signaling system is highly conserved in *Drosophila*. Different components of the fly insulin signaling pathway have strong homology with members of the vertebrate pathway^26^. In particular, some insulin-like peptides of flies (Dilps) share sequence, structural and functional similarities with vertebrate insulin, regulating glucose homeostasis^27^. Dilps and the glucagon analog adipokinetic hormone (AKH) are responsible for the maintenance of hemolymph glucose levels in insects^28^. Moreover, mutations in the components of the insulin pathway in flies result in reduced size of cells, organs, and bodies^25^. Similarly, the ablation of insulin-producing cells in the fly's brain produces decreased animal size and increased circulating sugar concentrations^29^.

In addition, like the insulin system, the mammalian JAK/STAT signaling pathway is also present in flies, and its role was described first in embryonic segmentation. The JAK/STAT route in flies consists of four main components. The ligand, cytokine-like Unpaired (Upd), the only receptor Domeless (Dome), JAK (Hopscotch/Hop), STAT (STAT92E), and transcriptional targets like socs36 and ptp61 are negative regulators of the pathway^30^.

Flies fed a high-fat or high-sugar diet develop phenotypes that model T2D, hyperglycemia, and IR^31–33^. In response to hypercaloric diets, the fat body (FB) secretes Upd2 (leptin like peptide), a cytokine that activates JAK/STAT signaling in GABAergic neurons in the brain. JAK/STAT activation allows the suppression of the inhibitory effect on insulin-producing cells (IPCs), thereby promoting the release of Dilps into general circulation, where they act on target tissue to stimulate fat storage and systemic growth^31^. Moreover, the exacerbated release of Drosophila Insulin-like Peptide 2 (Dilp2), functionally analogous to human insulin, promotes peripheral IR^33^. IR Drosophila models show an increase in circulating levels of both glucose and trehalose in the hemolymph of larvae and adults. Additionally, high expression levels for dilp 2, 3, 5 have been observed even when the level of sugar in the hemolymph remains high, similar to what occurs in mammals with glucose-insulin^25^. Furthermore, larvae that develop IR have higher stored and circulating TG levels^33,34^.

Our main results in this work are obtained from the fly FB organ that is considered functionally to be adipose and hepatic tissue, as it combines the capacities of energy storage, detoxification, and production of digestive enzymes, among others^35,36^. The organ is also involved in immune response and general metabolism as a nutritional sensor (reviewed in^37^). In this study, we propose that the JAK/STAT pathway plays a fundamental role in the regulation of glucose metabolism in *Drosophila*. In particular, we demonstrate that the loss of the dome receptor expression in FB reverses the effect of a high-sugar diet (HSD) and reduces IR in a T2D *Drosophila melanogaster* model.

## Materials and methods

### Fly stocks and diets

The Cg-gal4 (#7011), UAS-Dome-IR (#34618), UAS-mCD8-GFP (#5137), Canton-S (#64349) and 10XStat92E-GFP (#26197) stocks were obtained from Bloomington Drosophila Stock Center and maintained under standard conditions (25 °C, 50% relative humidity, 12/12-h light/dark cycles) in normal diet (ND, 0.15 M sucrose). For experiments, flies UAS-mCD8-GFP and UAS-Dome-IR were crossed to Cg-Gal4 > UAS-mCD8-GFP, and embryos were collected on apple agar plates for 24 h. Early first instar larvae were transferred to an ND or high sucrose diet (HSD, 1 M sucrose) for 72 h and 120 h respectively at 29 °C.

The diets were prepared using Sigma reagents according to protocols previously described in the literature^33^. For more details on the formulation of diets, review Supplementary Material Table 1.

### JAK/STAT activation and Nile Red Staining images

For determinate JAK/STAT activation, first instar larvae of 10XStat92E-GFP genotype were transferred to ND or HSD for 24, 48, or 72 h at 25 °C. FB tissue was dissected in PBS and fixed with 4% paraformaldehyde in PBS overnight at 4 °C. Then, the tissue was rinsed three times using PBST 0.3% (0.3% (v/v) Triton X-100 in PBS). Also, 2 ng/mL of DAPI was used to stain nuclei. Afterward, we mounted the stained samples in Vectashield (Vector Laboratory; H-1000) for microscopy analysis.

For lipid droplet staining, FB was dissected in PBS and fixed with 4% paraformaldehyde in PBS overnight at 4 °C. The tissue was rinsed three times with PBS, incubated for 2 h with 1 µg/mL Nile Red (Sigma, 7385-67-3) in PBS, and washed three times with PBT 0.1% (0.1% (v/v) Triton X-100 in PBS) and three times with PBS. DAPI (2 ng/mL) was used to stain nuclei. Then, stained samples were mounted in Vectashield for microscopy analysis. Images were acquired in a C2 + SiR Confocal microscope (Nikon Corporation, Tokyo, Japan) using NIS-elements program (Nikon Corporation, Japan). To quantify lipid droplet size, we measured the area of lipid droplets from ~ 50 fat body cells using Image J (NIH, USA, 1.52a).

### Real-time reverse transcription-polymerase chain reaction (qPCR)

RNA was isolated from whole larvae, brain, or FB using a Trizol (Ambion Life Technologies, CA, USA) according to manufacturer instructions. The cDNA was synthesized from RNA (2 mg) samples using the Applied Biosystems High-Capacity RNA-to-cDNA Kit (Applied Biosystems, CA, USA) according to standard procedures. The mRNA level expression analysis was performed by quantitative PCR, using the LightCycler96 instrument (Roche Diagnostics, Mannheim, Germany). The reaction was performed using 200 ng of cDNA with LightCycler FastStart DNA Master SYBR Green I kit (Roche, IN, USA) for a final volume of 10 mL. All reactions were performed in duplicate and negative controls were included. Gene expression was calculated using the model described by Pfaffl^38^ using rpl19 as a housekeeping agent. PCR primer sequences are shown in Supplementary Material Table 2.

### Body weight measurement

Larvae were collected and washed twice with PBS to remove any remaining food. After that, larvae were dried on absorbent paper and individually massed to obtain an average weight of ~ 20 larvae per condition in at least three independent experiments.

### Hemolymph glucose and lipid measurements

For hemolymph glucose measurement, we validated the use of the Accu-Chek Performa blood glucose meter (Roche, Mannheim, Germany). For this, we verifyied that the glucometer linearly discriminates glucose levels, for which we used both glucose (0.5 to 20 mM) and sucrose standard curve (0.05 to 1 M). These results show a linear relationship between the know concentration of glucose and quantification by Accu-chek glucometer (Fig. S1A). Moreover, measurements of concentrations lower than 0.5 M sucrose (11.399 mg/dL) gave the indication “low”, corresponding to a value lower than 10 mg/dL of glucose (detection limit). Values higher than 0.5 M sucrose, marked between 10 and 20 mg/dL, which accounts for the glucose specificity detection by glucometer (Fig. S1B). Subsequently, we performed a quantification assay on hemolymph samples obtained from a pool of hyperglycemic larvae (fed with HSD) of 10X-STAT92E-GFP genotype. We diluted the hemolymph in water (10–100%) to corroborate the linear determination of glucose present in the hemolymph. We demonstrated that the Accu-Chek glucometer measures hemolymph glucose as expected (Fig. S1C). Finally, we evaluate whether glucose determinations using the glucometer are comparable with determinations made with a commercial colorimetric assay compatible with different biological samples (Glucose (HK) Assay kit, #GAHK20, Sigma-Aldrich, USA), showing no difference between the two methods (Fig. S1D).

For glucose and lipid measurements in the different experimental conditions, hemolymph was extracted from ~ 70 larvae to obtain 10 µL for assay. For glucose measurement, 1 µL of hemolymph was quantified in Accu-Chek Performa blood glucose meter. Besides, to measure TAGs, 2 µL of hemolymph were analyzed with Triglyceride Quantification Colorimetric/Fluorometric Kit (Sigma-Aldrich, #MAK266) following the manufacturer instructions.

### Lifespan

We collected ~ 300 male flies for up to 24 h post-eclosion and placed in groups of 40 flies/vial for both ND and HSD groups to evaluate the effect of dome loss over the lifespan. The number of dead flies was counted every two days, and those that remained alive were transferred to a new vial with the corresponding diet. Statistical analysis was performed by comparing each population with the control.

### Statistical analysis

All data are presented as means ± standard error of the mean (SEM). We used a two-tailed t-test to compare diet effects of 10XSTAT92E-GFP and Canton-S genotypes. Additionally, we applied two-way ANOVA followed by Bonferroni's post-hoc test using GraphPad Prism software version 8.0.2 (GraphPad Software Inc.) for comparisons for the remaining experiments. A *p* value lower than 0.05 was considered statistically significant with *p ≤ 0.05, **p ≤ 0.01 and ***p ≤ 0.001.

## Results

First, we consider that the activation of JAK/STAT has shown to be a conserved mechanism of response to different stimuli, such as diet, infections, wounds, and stress. Previous reports show that diverse hypercaloric diets induce IR phenotypes, with high levels of circulating sugar and accumulation of TG in adipose tissue^33,34^. We thus examined the role of JAK/STAT signaling in the metabolic response that leads to the loss of homeostasis and IR in *Drosophila* larvae fed in a HSD.

### High-sugar diet induces the JAK/STAT pathway activation in fat body cells

**Figure 1:**
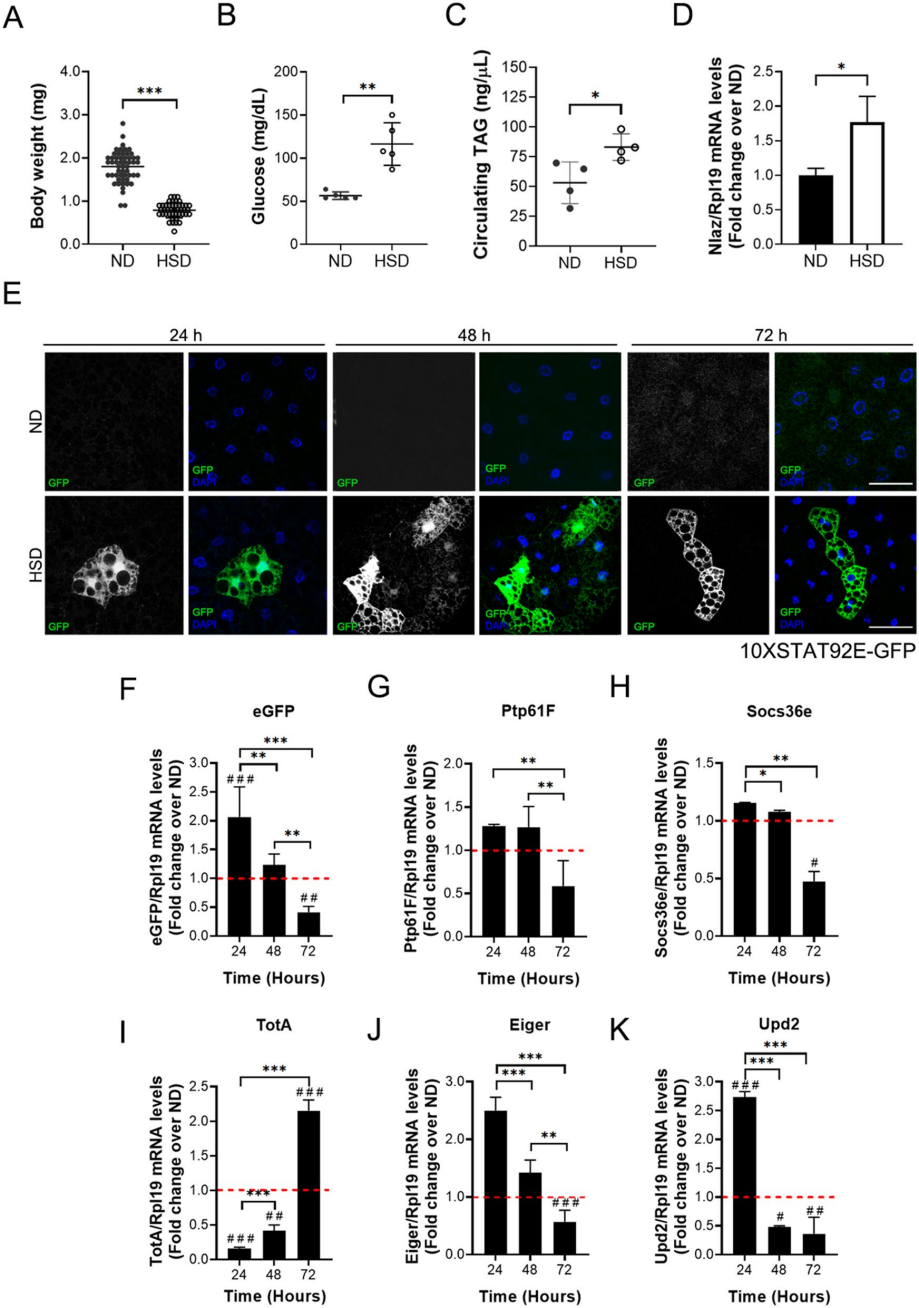
A high-sugar diet promotes JAK/STAT signaling activation in fat body cells. 10XSTAT92E-GFP larvae were reared on ND and HSDs. 72 h ND and 120 h HSD fed larvae (**A**) Bodyweight (n ≥ 20 animals at least 3 independent experiments), (**B**) Hemolymph glycemia (n = 70 larvae per pool, N ≥ 3 independent experiments), (**C**) Hemolymph TAG levels (n = 70 larvae per pool, N ≥ 3 independent experiments) and (**D**) Relative expression by qPCR to Nlaz in fat body cells (n = 25 larvae per pool, N ≥ 3 independent experiments). Bars represent mean ± SD. An unpaired two-tailed t-test was used to derive *p* value. ***p < 0.001, **p < 0.01, *p < 0.05 (**E**) Representative images of 10XSTAT92E-GFP reporter activations in fat body cells at 24, 48 and 72 h in 10XSTAT92E-GFP fat body larvae fed with normal (ND) or high-sugar diet (HSD). The panel shows cells marked with DAPI (blue), which expressed eGFP (green) when JAK/STAT signaling was activated. Scale bar = 50 µm. Relative expression by qPCR to ***eGFP*** (**F**), *ptp61F* (**G**), *socs36E* (**H**), *totA* (**I**), *eiger* (**J**), and *upd2* (**K**) in fat body cells at 24, 48, and 72 h in larvae fed with an ND or HSD. The dotted line (red) represents the relative expression on larvae fed with ND (n = 25 animals per group, N = 3 independent experiments). 2-way ANOVA followed by Bonferroni's multiple comparisons test was used to derive all *p* values. ***p < 0.001, **p < 0.01, *p < 0.05. ^###^*p* < 0.001, ^##^*p* < 0.01, ^#^*p* < 0.05 respect to ND control.

To determine the impact of a HSD in JAK/STAT pathway activation in the FB cells, we used the Stat92E-responsive transcriptional 10xStat92E-GFP fly reporter, which provides an accurate representation of endogenous pathway activity^39^. Initially, we demonstrated that 10xStat92E-GFP larvae replicate the insulin resistance phenotype caused by HSD. Larvae fed with HSD show a lower body weight (Fig. 1A), accompanied by an increase in glucose (Fig. 1B) and TAG (Fig. 1C) hemolymph levels in comparison to larvae fed a ND. Additionally, we evaluated Lipocalin-encoding Neural Lazarillo (Nlaz) expression in the FB cells, which is a lipocalin induced by JNK in response to HSD in the FB, and it is considered to be an IR marker^40^. The overexpression of Nlaz provokes IR in peripheral tissues, and the loss of its expression in the FB protects larvae from this condition^40^. We show that the HSD induces a higher expression of Nlaz in the 10XStat92E-GFP larvae (Fig. 1D). Concordantly, the results of weight, glycemia, circulating TAGs and Nlaz expression confirms that the 10XStat92E-GFP larvae reproduces an IR phenotype, induced by a HSD.

Moreover, we observed that a HSD activated the JAK/STAT pathway in the FB cells, revealed by the eGFP signal compared to ND fed larvae (Fig. 1E). This result is consistent with *eGFP* mRNA expression at 24 h (Fig. 1B). Furthermore, we analyzed the transcriptional activation of Stat92E targets, *ptp61F*, *socs36e*, repressors of the pathway, and *totA* by a HSD. The decrease in expression of *ptp61F* and *socs36e* (Fig. 1C,D) and induction of *totA* (Fig. 1E) at 72 h confirmed that the JAK/STAT pathway is activated in a lifespan larvae feeding with HSD. Additionally, we observed an increased expression of *eiger* (TNF-α) (Fig. 1F), a pro-inflammatory cytokine involved in several aspects of insulin signaling inhibition^41,42^. Previous reports show that the FB secreted TotA under different stress conditions, such as immune challenge and environmental stress^43,44^. Therefore, an increase in TotA and Eiger expression supports the idea that a HSD is an inflammatory signal in FB tissue.

Consequently, in response to a HSD, the FB increased the expression of *upd2*, a cytokine related to insulin signaling, that promotes the secretion of Dilp2 by IPCs^31^. We determine that a HSD induces an early response in mRNA expression of *upd2* which decays at 72 h (Fig. 1G), suggesting a decrease in the secretion of Dilp2 and a deficient insulin signaling.

### Dome loss in fat body cells reverts insulin resistance phenotypes induced by a high-sugar diet

**Figure 2:**
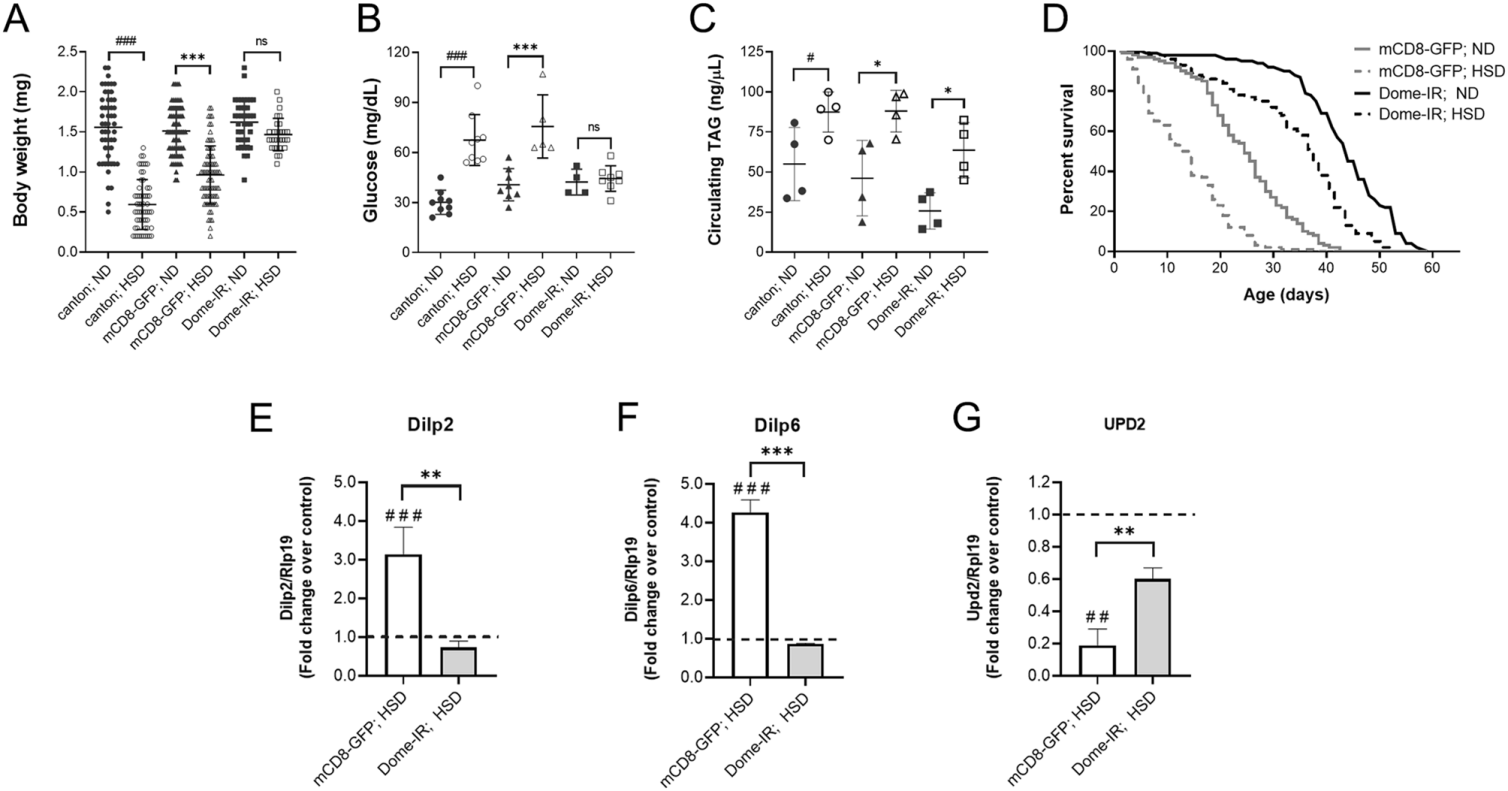
Specific *dome* knockdown in fat body cells reverses the effects of a high-sugar diet. Canton-S, control (mCD8-GFP), and Dome knockdown (Dome-IR) larvae were reared on ND for 72 h and HSD for 120 h. (**A**) Bodyweight (n ≥ 30 animals at least 4 independent experiments), (**B**) Hemolymph glycemia (n = 70 larvae per pool, N ≥ 3 independent experiments) and (**C**) Hemolymph TAG levels (n = 70 larvae per pool, N ≥ 3 independent experiments) of larvae fed with ND or HSD. (**D**) Lifespan curve of Dome-IR and mCD8-GFP adult male flies fed a ND or HSD (n = 300 animals per conditions). Relative expression by qPCR to *dilp2* in brain cells (**E**), *dilp6* (**F**), and *dilp2* (**G**) in fat body cells of Dome-IR and mCD8-GFP larvae in HSD (n = 25 per group, N = 3 independent experiments). The dotted line (black) represents the relative expression by qPCR of mCD8-GFP and Dome-IR larvae fed with ND. Bars represent mean ± SD. 2-way ANOVA followed by Bonferroni's multiple comparisons test was used to derive all *p* values. ***p < 0.001, **p < 0.01, *p < 0.05. ^###^*p* < 0.001, ^##^*p* < 0.01, ^#^*p* < 0.05 respect to ND control.

To determinate the role of JAK/STAT signaling in the FB of larvae fed with a HSD, we used a transgenic line that expressed RNAi in the FB (Cg-Gal4, UAS-Dome-IR) and that provokes a reduced expression of Dome in this tissue^45^. Specifically, *dome* knockdown reached 70% under our conditions, evaluated as mRNA levels by qPCR (Fig. S2A). These larvae, submitted to HSD, displayed a lower feeding rate (Fig. S2B), although their sugar intake is still higher. Additionally, the same behavior is observed in wild type larvae, Canton-S (Fig. S2C). Moreover, the FB-specific knockdown of *dome* was able to reverse the HSD effects on body weight and glycemia levels compared to control larvae raised on a ND (Cg-Gal4, UAS-mCD8-GFP) (Fig. 2A,B). Besides, Dome’s loss in the fat body partially reverses the increase in circulating trehalose levels induced by HSD (data not shown). Furthermore, we do not observe a decrease in circulating TAGs levels (Fig. 2C).

It has been previously described that the JAK/STAT pathway is involved in many aging-related functions, including cell proliferation, differentiation, survival, apoptosis, and cell senescence^46,47^. Furthermore, the activation of JAK/STAT in the FB cells by hemocytes-derived Upd3 causes a shorter lifespan in response a HFD^32^.

In our model, control larvae fed with a HSD showed a 44% reduction in the median lifespan compared to larvae raised with a ND (Fig. 2D). Additionally, we showed that the Dome receptor´s loss in the FB cells increased the median lifespan by 76% and 52% in larvae raised on a ND and HSD, respectively (Fig. 2D). These results suggest that the JAK/STAT pathway plays a similar role in response to both hypercaloric diet types (HFD and HSD) in animal longevity.

In the context of IR, it has been demonstrated that an increase in expression and accumulation of Dilp2 in the IPCs, depends on Dilp6 and Upd2 fat body signaling^31,47,48^. Dilp6 is an insulin like-peptide secreted from the FB tissue, and it is an inhibitor of the release of Dilp2 to the hemolymph^48^. As expected, we observed an increase in the expression of *dilp2* in brain cells of larvae fed with a HSD (Fig. 2E). This result was accompanied by a rise in the FB cells’ *dilp6* expression levels (Fig. 2F) and a decrease of *upd2* (Fig. 2F). Previously, we showed that *upd2* is activated at 24 h and decreases its expression at 48 and 72 h in HSD compared to ND (Fig. 1K), indicating that in lifespan larvae fed with HSD, the FB cells do not produce *upd2* after 48 h. The diminished *upd2* and increment of dilp6 expression is consistent with the transcription activation of *dilp2* in the IPCs and suggest a delivery failure to Dilp2 to hemolymph. Further, in agreement with the reversion in body weight and glycemic levels, Domeless receptor loss in the FB cells reverted the effect of a HSD on *dilp2*, *upd2*, and *dilp6* expression in the brain and the FB, respectively (Fig. 2). Our results support the idea that loss of Dome in the FB counteracts the effects of IR.

### Dome receptor loss counteracts the effects of a high-sugar diet on lipid metabolism in fat body cells

**Figure 3:**
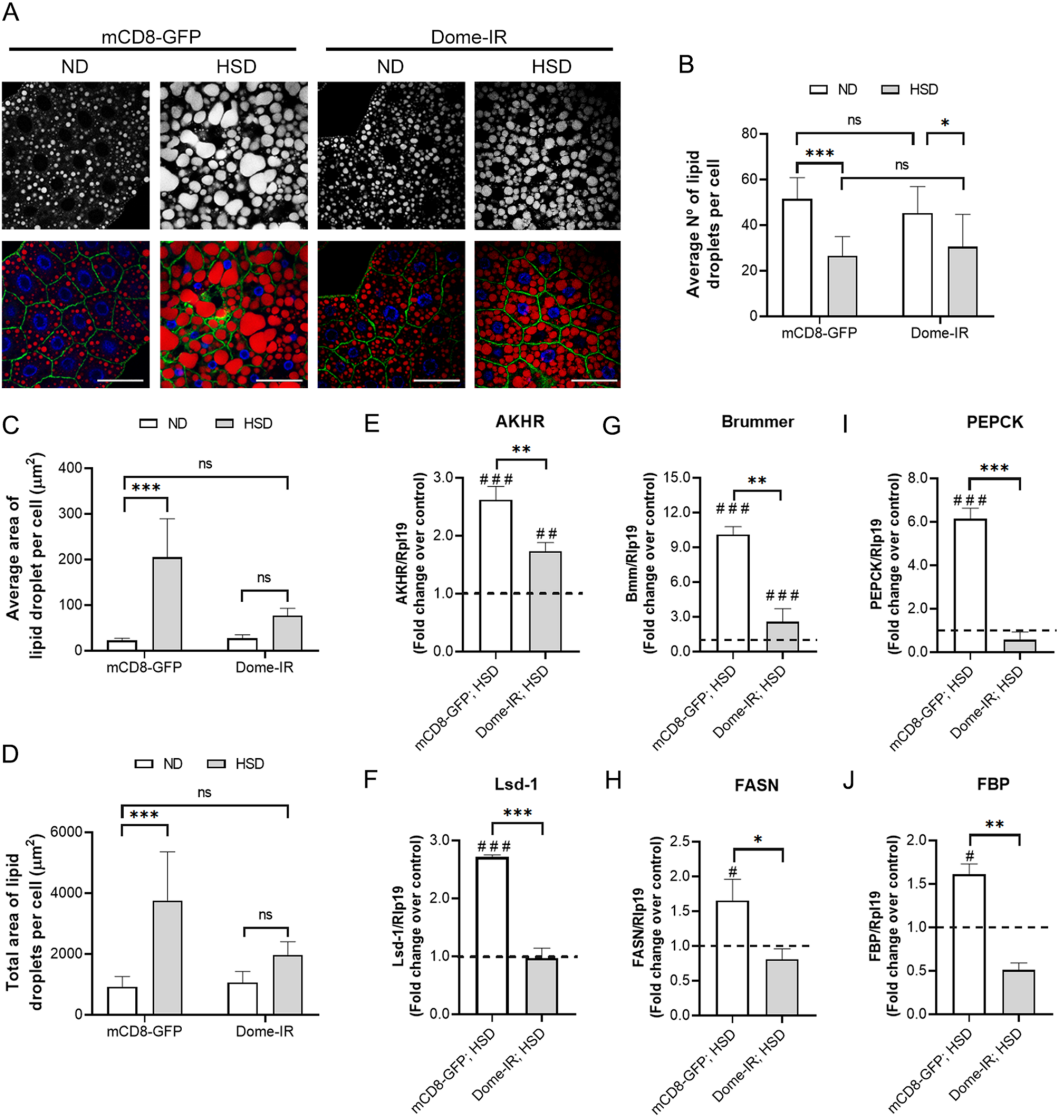
Dome loss in fat body cells reverses HSD effects on synthesis or lipid store. Control (mCD8-GFP) and Dome knockdown (Dome-IR) larvae were reared on ND for 72 h and HSD for 120 h. (**A**) Representative image of lipid droplets in fat body cells in different experimental conditions. The panel shows plasmatic membrane by mCD8-GFP expression (green), Nile Red stain (red), and DAPI (blue). Scale bar = 50 µm. (**B**) Quantification of the number of lipid droplets per cell**,** (**C**) average area of lipid droplets, and (**D**) total area of lipid droplets per cell in Dome-IR and mCD8-GFP early third instar larvae in normal or high-sugar diets. (n = 12 animals per group, N = 3 independent experiments). Relative expression by qPCR to *akhr* (**E**), *lsd-1* (**F**), *Bmm* (**G**)**,** *fasn* (**H**), *pepck* (**I**), and *fbp* (**J**) in fat body cells of Dome-IR and mCD8-GFP larvae fed in HSD (n = 25 animals per group, N = 3 independent experiments). The dotted line (black) represents the relative expression by qPCR of mCD8-GFP and Dome-IR larvae fed with ND. Bars represent mean ± SD. 2-way ANOVA followed by Bonferroni's multiple comparisons test was used to derive all *p* values. ***p < 0.001, **p < 0.01, *p < 0.05. ^###^*p* < 0.001, ^##^*p* < 0.01, ^#^*p* < 0.05 respect to ND control.

In a HSD, elevated lipogenesis protects the cell against caloric overload, it produces TAG storage, an increase in the size of lipid storage droplets, and a decrease in the number^33^. To determine the role of the JAK/STAT pathway in lipid storage, we analyzed the distribution and lipid content in the FB cells, staining TGs in lipid droplets (LD) with Nile Red. As shown in Fig. 3, the Dome-IR FB the cells showed a lower number of LD, which were smaller in size compared to observed in the FB cells of control larvae with a HSD. Thus, Dome loss restored lipid content in the larvae´s FB cells fed a HSD (Fig. 3A–D).

Additionally, as a part of the alterations established during IR, changes in the expression of proteins related to glucose metabolism, lipid storage and mobilization are observed in adipose tissue. For example, the AKH/AKHR signaling is involved in developing hyperglycemia under HSD conditions (Reviewed in ^23^). Moreover, it stimulates the expression of lipolysis related proteins, such as lipid storage droplet-1 (Lsd-1), Brummer (Bmm)^49^, and also enzymes that participate in gluconeogenesis (PEPCK and fructose-1,6-bisphosphate (FBP)^50^, which explains the hyperglycemia produced by a HSD. Considering that the JAK/STAT pathway affects TAGs content in FB, we analyzed the expression of AKHR, lipid metabolism, and gluconeogenesis-related enzymes in Dome-IR larvae fed a HSD or ND. We observed that a HSD induced the expression of genes related to both lipolysis (*akhr, bmm, and lsd1*; Fig. 3E–G), lipogenesis (*fasn*, Fig. 3H) and gluconeogenesis (*pepck* and *fbp*, Fig. 3I,J). It could reflect the mobilization and storage of lipids simultaneously^51^. This metabolic phenomenon has been described previously as an effect of HSD^33,34^. However, *dome* knockdown in the FB cells significantly reduced this effect in the expression of lipid and carbohydrate metabolism genes near the control level (Fig. 3E–J). Also, the balance in the expression of *lsd1* (Fig. 3F), *bmm* (Fig. 3G), and f*asn* (Fig. 3H) could explain the lesser storage and content of TAGs in the FB cells (Fig. 3B–D).

### JAK/STAT pathway in fat body cells responds to the IR phenotype in *D. melanogaster* larvae

**Figure 4:**
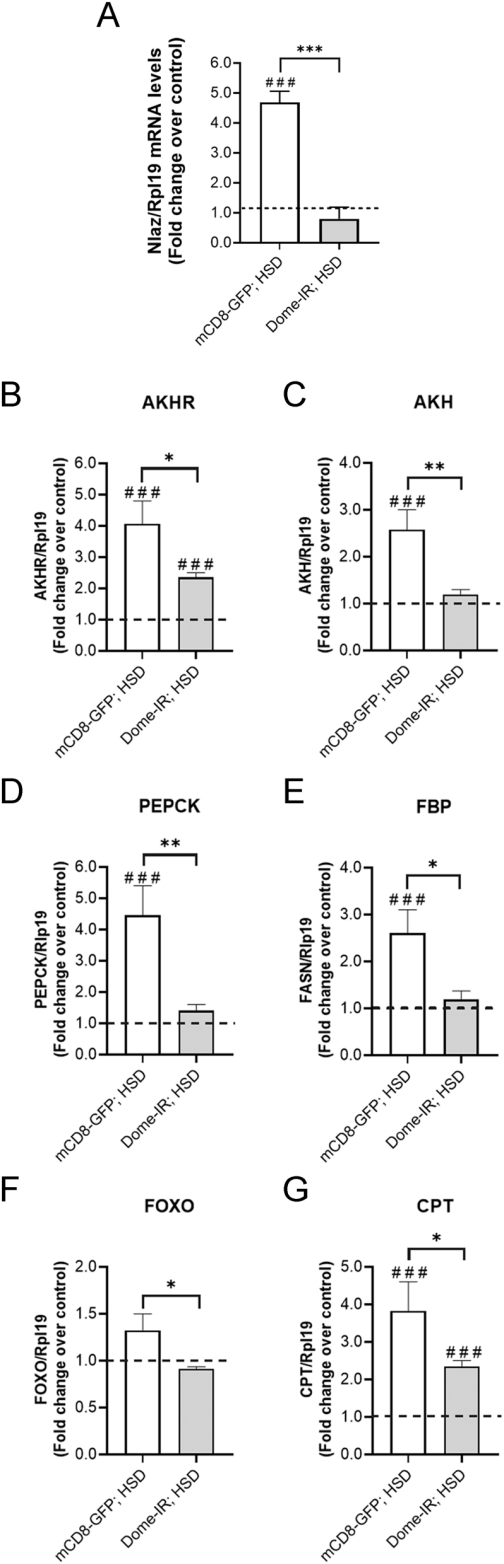
Dome loss in fat body reversed high-sugar diet effect on peripheral insulin resistance related-markers. Control (mCD8-GFP) and Dome knockdown (Dome-IR) larvae were reared on ND for 72 h and HSD for 120 h. Relative mRNA levels evaluated by qPCR to Nlaz (**A**) in fat body cells, lipid mobilization related-proteins AKHR (**B**) and AKH (**C**), gluconeogenesis related-enzymes, PEPCK (**D**) and FBP (**E**) and FOXO signaling proteins, FOXO (**F**) and CPT (**G**) in whole larvae tissue of Dome-IR and mCD8-GFP early third instar larvae fed with normal diet or a high-sugar diet (n = 25 animals per group, N = 3 independent experiments). The dotted line (black) represents the relative expression by qPCR of mCD8-GFP and Dome-IR larvae fed with ND. Bars represent mean ± SD. 2-way ANOVA followed by Bonferroni's multiple comparisons test was used to derive all *p* values. ***p < 0.001, **p < 0.01, *p < 0.05. ^###^*p* < 0.001, ^##^*p* < 0.01, ^#^*p* < 0.05 respect to ND control.

Several high-sugar-induced changes in mRNA levels of genes involved in lipid and glycolytic metabolism in peripherals tissues occur. These with the overexpression of Nlaz in FB cells indicate IR in the *Drosophila* T2D model^40^. The dysregulation in lipid mobilization mediated by the overexpression of AKH/AKHR has been previously described for IR, as well as the activation of gluconeogenesis and FoxO (an insulin-dependent transcription factor). This information is coincident with the upregulation of lipogenesis and trehalose synthesis^33^. We demonstrated that Dome loss reversed the effect of the HSD over Nlaz in the FB cells (Fig. 4A), decreasing the expression of *akh* and *akhr* (lipid mobilization) (Fig. 4B,C), and also *pepck* and *fbp* (gluconeogenesis) (Fig. 4D,E) in peripheral tissues. Additionally, *dome* knockdown diminished the expression of *foxo* and its target, Carnitine palmitoyltransferase (*cpt*) (Fig. 4F,G). In mammals, FoxO proteins promote TAG catabolism in adipose tissue by stimulating the expression of adipose TAG lipase (ATGL). As a whole, these results suggest that JAK/STAT activation in the FB cells induced by a HSD alters lipid and glucose metabolism, as well as insulin signaling. Hence, it promotes IR in peripheral tissues, consistent with the IR phenotype described in a previous study^33^.

## Discussion

The main finding of the present study was that the knockdown of the JAK/STAT receptor, *domeless*, in the FB tissue protects from developing an IR condition induced by a HSD in *D. melanogaster* larvae. In addition, associated with the inhibition of the JAK/STAT pathway, we observed attenuation in the increased expression of key enzymes of lipid and glucose metabolism observed in a T2D model, leading to a normal lipid accumulation in the FB.

Excess caloric intake has been associated with a variety of human pathologies. In particular, diets high in sugar induce various pathological states strongly linked to the development of diseases such as obesity, metabolic syndrome, and T2D^11,52^. Additionally, it has been proposed that T2D patients have a chronic state of inflammation that is expressed by a high level of circulating cytokines^53,54^. Moreover, TNF-α and IL-6 can directly alter insulin sensitivity by activating different signaling pathway steps^16,55^. For example, these cytokines can stimulate phosphorylation of serine instead of tyrosine in the insulin-1 receptor (IRS-1), thereby inhibiting the activation of insulin signaling and emphasizing IR^56^. Besides, hyperglycemia also induces the production of IL-6 from endothelium and macrophages, thus generating a vicious cycle. Thus, hyperglycemia improves the action of the cytokine signaling suppressor (SOCS), altering the release of insulin and its signaling pathway. The SOCS family of proteins can also inhibit the activity of JAK signaling transducers and STAT transcription activators in various tissues^55^. The JAK/STAT dysregulation in adipocytes and its contribution to the development of obesity and diabetes has been demonstrated in various models. Adipose tissue inflammation in obese patients promotes STAT1 activation to induce IR by blocking Akt phosphorylation and expression of the insulin receptor, IRS1, and glucose transporter type (GLUT) 4^57^. This finding suggests that improving glycemic control could reduce the inflammatory response that supports the link between inflammation and glucose metabolic disorders.

In the search for models to analyze the fundamental metabolic adaptations of T2D induced by a hypercaloric diet, *D. melanogaster* arose as one of the most reliable systems. *Drosophila* is a straightforward model that displays a low functional redundancy that allows the study of activation of specific routes with minimal interference^58^. *Drosophila* presents a model system for evaluating how different hypercaloric diets impact tissue growth regulation, glucose homeostasis, lipid metabolism, and longevity. Moreover, it has been demonstrated that changes in diet consumption have direct consequences for the developmental pattern of flies^33^. In this sense, not only the caloric overload plays a role, but also the type of substrate (type of sugar, for example)^33,59^ and the relationship between macronutrients (protein–sugar–fat ratio)^60^. Additionally, the effects of diet can exert transgenerational changes, modulating the size, reproductive capacity and survival of the offspring^61^. The effects of a diet rich in calories, particularly rich in sugar, are metabolically manifested in energy storage^33,59^. Trehalose, triglycerides, and glycogen are macromolecules that can be synthesized in response to the intake of hypercaloric diets and whose metabolism is directly related to mechanisms that determine the development of pathophysiological conditions^33,59^.

Due to the multifunctionality of the fat body in flies, which allow this organ to respond various of stimuli and play a prominent role in the regulation of larval size and development, we studied the participation of the JAK/STAT signaling pathway in the regulatory response to a HSD. It is important to note that the *Drosophila* genome encodes for only three related JAK/STAT ligands, Upd, Upd2, and Upd3^62^. In this sense, previous reports showed that septic injury induces Upd3 secretions by hemocytes (fly macrophages) and causes JAK/STAT activation in the FB, which results in the expression of the stress response factor TotA^43^. Similarly, in our work, we found that the consumption of a HSD induced an early Upd2 and Eiger expression, and it was accompanied by the JAK/STAT activation in the FB with a late expression of TotA (Fig. 1). These results are also reflected in the triglyceride content as a decrease in the number and size of lipid droplets, suggesting that the JAK/STAT pathway activation in the FB is involved in the inflammatory response that has been linked previously to the development of insulin resistance^63^.

Furthermore, it seems to us that Dome receptor loss in FB cells contributed to a reversion to the normal phenotype and improved the metabolic response to a HSD. Specifically, *dome* knockdown attenuated the effect of a HSD on body weight and glucose level in hemolymph, indicating that inhibition of *dome* expression could revert the insulin resistance phenotypes (Fig. 2A,B). Consistent with these findings, we observed that Dome loss diminished the expression of inflammatory markers increased in a HSD due to IR. This is reflected in the reduction of the expression of *dilp6* and *upd2* in the FB cells and *dilp2* in brain cells (Fig. 2D–F).

Besides, a study showed that flies fed a lipid-rich diet expressed higher levels of *upd3* in hemocytes. This cytokine was responsible for the reduction in lifespan through global JAK/STAT activation^32^. Additionally, it was shown that the mere fact of ectopically overexpressing *upd3* in both hemocytes and the FB reduced fly lifespan fed a control diet^32^. In the same study, authors observed that Upd3 null flies did not develop hyperglycemia and did not show a JAK/STAT activation in response to a lipid-rich diet, having an equal lifespan to flies fed in a normal diet. In our work, we showed that even though flies fed a HSD had a reduced lifespan compared to those fed a control diet, Dome receptor loss in the FB not only improved the lifespan of flies fed a HSD but considerably extended the lifespan of flies fed a control diet (Fig. 2C). Given our results, it is possible to consider that JAK/STAT activation in the FB is responsible for the lifespan effects in flies fed a HSD.

In mammals, under inflammatory conditions associated with obesity, STAT1 can decrease the hydrolysis of circulating triglycerides and their storage in adipocytes by inhibiting the expression of lipoprotein lipase^21^. Conversely, in mice that are deficient for STAT4 in adipocytes and fed a high-fat diet, better insulin response and better glucose homeostasis are observed^64^. Furthermore, JAK/STAT signaling modulates key enzymes involved in lipogenesis, such as acyl CoA oxidase (AOX), the rate-limiting enzyme in peroxisomal fatty acid β-oxidation and fatty acid synthase (FASN)^65^. Challenging the idea that obesity can cause T2D, alternative evidence suggests that an increase in fat storage in adipose tissue protects mice from T2D^66,67^. In this sense, animals that cannot store fat normally, are insulin-resistant when fed normal diets^68,69^.

Consistently, ectopic lipid accumulation in non-adipose tissues such as muscle, liver, or blood is associated with IR^70^. In this work, we associated the JAK/STAT activation with energy overload managed through lipid metabolism in the FB of *Drosophila* fed a HSD. We show that Dome receptor loss reverted the stimuli on the expression of storage and lipid mobilization related-proteins (Lsd-1 and Bmm) induced by a HSD, indicating that the JAK/STAT regulates lipid metabolism (Fig. 3E,F). These results are also reflected in the triglyceride content, as a decrease in the number and size of lipid droplets (Fig. 3A–D). The stimulatory effect of a HSD in the expression of genes encoding gluconeogenic enzymes has been reported previously in mice^71^ and T2D patients^72^. Additionally, here we show that *dome* knockdown in the FB also reduced the effect of a HSD on the expression of the gluconeogenic enzymes PEPCK and FBP (Fig. 3G,H).

In starvation conditions, acute TG lipolysis is induced in *Drosophila*, which is attenuated in the FB cells of AKHR mutant flies. Also, in vitro and in vivo studies have identified that Lsd-1 (PLIN1) is a PKA phosphorylation target that has a central role as a pro-lipolytic effector of the AKH/AKHR pathway on lipid droplet surface^73^. Our results show that Dome loss in the FB significantly reduced the effect of a HSD on AKHR and FASN expression, which may explain the decrease in TGs storage and a low expression of *lsd-1*, despite the energy oversupply of a HSD (Fig. 3I,J).

The obesity and IR model in *Drosophila* was first described in 2011^33^. Authors elegantly showed how a HSD induces IR and how the mechanism by which *Drosophila* responds to a diet rich in carbohydrates is highly conserved in different animals. In this model, the response to a HSD involves activating the same signaling pathways that lead to IR, seen in *C. elegans*, mice and humans^33^. In particular, a HSD induces transcriptional changes that account for IR at the peripheral level. Different key molecules of both carbohydrate and lipid metabolism are up-regulated in response to a HSD^33^.

Additionally, the FoxO transcription factor that regulates IR response and some of its transcriptional targets are also strongly altered^33,74^. Here we show how Dome loss in FB reduces the expression of various markers of IR that are significantly stimulated by a HSD in peripheral tissues (Fig. 4). Since our results show that Dome loss reduces stored lipids and improves glucose levels in a HSD, we think that JAK/STAT is part of the adaptive response, which regulates, by AKHR signaling, the balance between lipid and carbohydrate metabolism in response to dietary excess.

Finally, we propose that JAK/STAT, a signaling pathway that responds specifically to inflammatory stimuli, may constitute a possible target for future therapeutically-oriented interventions for IR and its consequences.

## Supplementary Information

Supplementary Information.

## Abbreviations

T2D: Type 2 diabetes
IR: Insulin resistance
FB: Fat body
Dome: Domeless
Nlaz: Lipocalin Neural Lazarillo
TNF-α: Tumor necrosis factor alpha
IL-6: Interleukin-6
Dilps: Insulin-like peptides
AKH: Adipokinetic hormone
Upd: Unpaired
IPCs: Insulin producing cells
HSD: High-sugar diet
HFD: High-fat diet
ND: Normal diet
FBP: Fructose-1,6-biphosphatade
PEPCK: Phosphoenolpyruvate carboxykinase
AKHR: Adipokinetic hormone receptor
TG: Triglycerides
Bmm: Brummer
LD: Lipid droplets
Lsd-1: Lipid storage droplet-1
FASN: Fatty acid synthase
FOXO: Forkhead box protein O
CPT: Carnitine palmitoyltransferase
IRS-1: Insulin-1 receptor
SOCS: Cytokine signaling suppressor
