# Supplementary material for: Domeless receptor loss in fat body tissue reverts insulin resistance induced by a high-sugar diet in Drosophila melanogaster
Source: Sci Rep. 2021 Feb 5;11:3263. doi: 10.1038/s41598-021-82944-4 (PMC7864986; doi:10.1038/s41598-021-82944-4)
Supplement: Supplementary file 1 — Supplementary Information. [file 41598_2021_82944_MOESM1_ESM.pdf]

Domeless receptor loss in fat body tissue reverts insulin resistance induced by a high-sugar diet in *Drosophila melanogaster*.

Fernanda Lourido, Daniela Quenti, Daniela Salgado-Canales and Nicolás Tobar

Supplementary Materials.

Supplementary Table 1. Nutrient content of control and high sugar foods. We use a formulation previously[33] a control diet and a high sugar diet that contains sucrose to replace glucose, based on Bloomington's semi-defined food.

Control Diet	100 ml		carbs (g)	protein (g)	fat (g)	
Agar	1.0g		0.89	0	0.01	
Brewer's Yeast	8.0g		3.2	3.6	0.08	
Yeast Extract	2.0g		0.33	1.08	0	
Peptone	2.0g		0.01	1.46	0	
Sucrose	5.1g		5.1	0	0	
$MgSO_4$ x $6H_2O$	0.05g					
$CaCl_2$ x $2H_2O$	0.05g					
Propionic acid			0.6g			
Mold inhibitor			1.1g			
						Total kcal
Carbs %		63.9%				
Protein %		34.9%				
Fat %		1.1%				
Total Grams			11.23	6.14	0.09	
Total Kcal			44.92	24.56	0.77	70.28

High-Sugar Diet	100 mL		carbs (g)	protein (g)	fat (g)	
Agar	1.0g		0.89	0	0.01	
Brewer's Yeast	8.0g		3.2	3.6	0.08	
Yeast Extract	2.0g		0.33	1.08	0	
Peptone	2.0g		0.01	1.46	0	
Sucrose	34.2g		34.2	0	0	
$MgSO_4$ x $6H_2O$	0.05g					
$CaCl_2$ x $2H_2O$	0.05g					
Propionic acid			0.6g			
Mold inhibitor			1.1g			
						Total kcal
Carbs %		86.4%				
Protein %		13.2%				
Fat %		0.4%				
Total Grams			40.33	6.14	0.09	
Total Kcal			161.32	24.57	0.77	186.66

Supplementary Figure #1. Validation of Accu-chek Performa glucometer for determining glucose in the hemolymph.

We verified that the glucometer linearly discriminates glucose levels as well as its specificity. The results obtained show that the determination of glucose levels using a standard glucometer does not differ significantly from those obtained with the colorimetric kit.

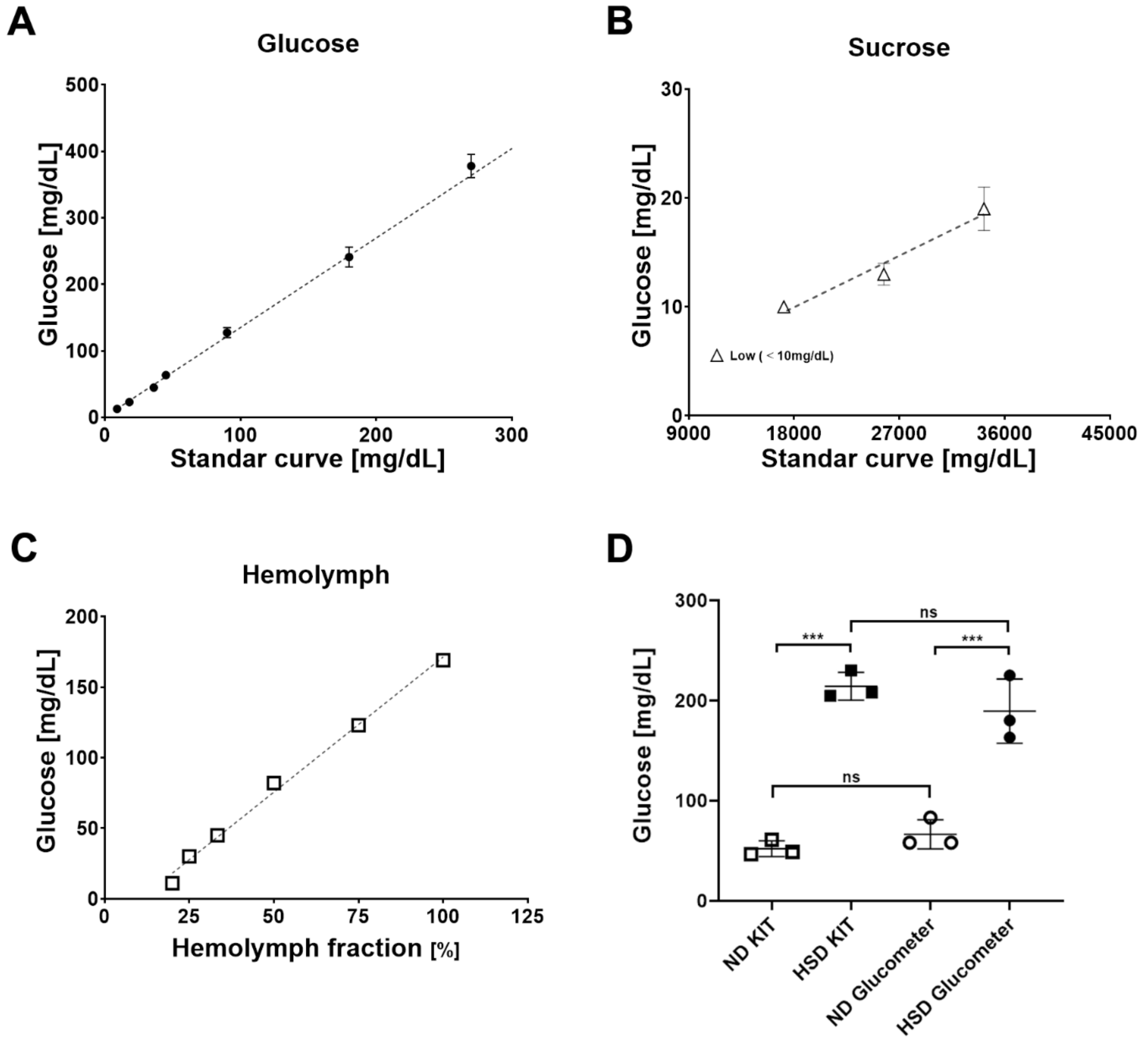

Supplementary Figure 1. The glucometer makes it possible to quantify glucose levels in the hemolymph. **A.** Glucose determination with a glucometer on a standard glucose curve (0.5 to 20 mM). **B.** Glucose determination with a glucometer, on a standard sucrose curve (0.05 to 1 M). **C.** Glucose determination with a glucometer, in a hemolymph dilution (10-100% in water) from larvae fed with HSD. **D.** comparison between glucose levels determined with a colorimetric kit (# GAHK20, Sigma-Aldrich, USA) and Accu-chek Performa glucometer in the same paired sample (hemolymph pool) from larvae fed with ND and HSD. The dotted line corresponds to the best linear fit of the points. (***) represents a statistically significant difference. One-way ANOVA, Tukey's multiple comparisons test.

Supplementary Figure #2

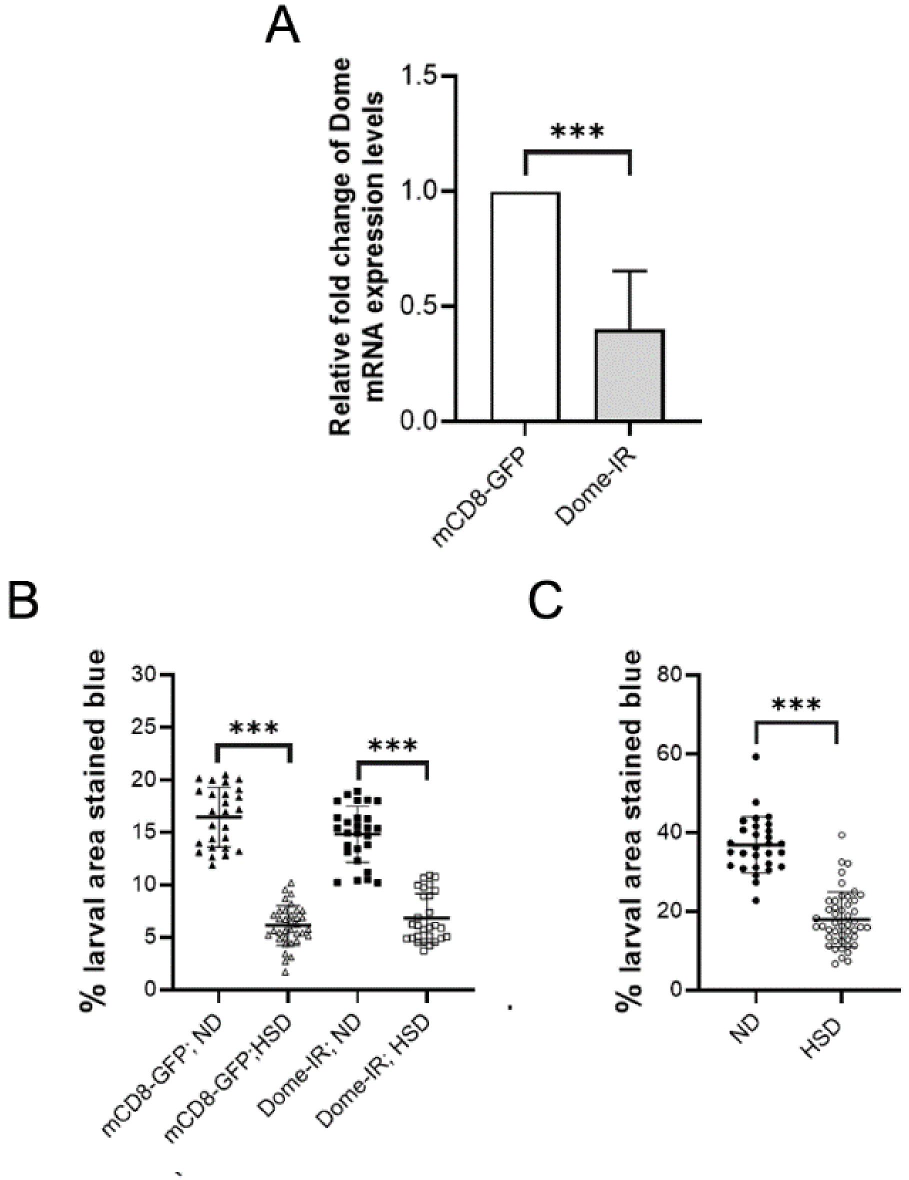

Supplementary Figure 2. A. Relative expression by qPCR to dome in fat body cells of mCD8-GFP and Dome-IR larvae fed with ND. (n = 25 larvae per pool, N≥3 independent experiments). Bars represent mean ± SD. An unpaired two-tailed t-test has been used to derive P-value. ***p<0.001, **p<0.01, * p<0.05. **B.** Percent of larval area stained with brilliant blue dye of mCD8-GFP and Dome-IR larvae fed with ND for 72 h and HSD for 120 h (n=10 larvae per pool, N=3). 2-way ANOVA followed by Bonferroni's multiple comparisons test was used to derive all P-values. ***p<0.001, **p<0.01, * p<0.05. **C.** Percent of larval area stained with brilliant blue dye of Canton-S larvae fed with ND for 72 h and HSD for 120 h. (n=10 larvae per pool, N=3). An unpaired two-tailed t-test was used to derive P-value. ***p<0.001, **p<0.01, * p<0.05.

Supplementary Table # 2: Primer list used for qPCR.

**Gen**	**Forward 5´→3´**	**Reverse 5´→3´**
Dome*	TCTGTCTGTGAACGCCACTC	TATCGAACCCTCGCATTCCG
eGFP[1]	AGATCCGCCACAACATCGAG	TCGTTGGGGTCTTTGCTCAG
NLaz*	GGACAACCCTCGAATGTAACT	GACGGCGTATGACTCGTAATC
TotA**	TTCCGACGAAGATCGTGAGG	CTGGGTGCTATTGATTTGGAGT
Socs36e*	CTGGGTGCTATTGATTTGGAGT	CGAGGATGTGGATGTGGAC
Ptp61F**	AACGGCATCGATCCAATTC	CCGCTTCAGCTCGTTCTC
Upd2[2]	AGTGCGGTGAAGCTAAAGACTTG	GCCCGTCCCAGATATGAGAA
Eiger*	CAGTGCATCCTCAGCCTCAA	GCAGGGCTCTCTTTGGAAGT
Dilp2[3]	CTCAATCCCCTGCAGTTTGT	CGCAGAGCCTTCATATCACA
Dilp6[4]	CGATGTATTTCCCAACAGTTTCG	AAATCGGTTACGTTCTGCAAGTC
AKHR**	GCTATCCACGGACCTGATGTG	CTGTCGAGCGATATGCAGACC
FBP**	CCGCCATCAAGGCTACATCAT	TTGGAGAGCACGTCCAGTTTC
PEPCK**	TGATCCCGAACGCACCATC	CTCAGGGCGAAGCACTTCTT
BMM*	CTGCTGTCTCCTCTGCGATT	TGTAGACCCTCCAGCAGACA
Lsd-1*	TTGGTTTCGGCCCTGAATGA	GGGAGAAGCGTTGACCATGA
FASN*	CCCGCTCCACTCCAAGAACT	TTCTGGGTGCTGGGTGAATG
AKH*	GGGAGAAGCGTTGACCATGA	AGCAGCATTTCGTTGGAGGT
Hex C**	CAGGCCAACGTCAAGTGTTTT	GTGGTGACCTTTCAGCGAGA
GAPDH[5]	CATTGTGGGCTCCGGCAA	CGCCCACGATTTTCGCTATG
FOXO**	CATGGGGAAATCTATCCTATGCG	ACTCAGTGTCAATCGTTTGTCG
CPT*	CGAGACCTTACCTTCGCTCC	TGGAACTCATCAATAGTCTTGCG

* Designed by NT's Lab

** Validated in https://www.flyrnai.org/cgi-bin/DRSC_primerbank.pl

1 Tan MC, Wong WY, Ng WL, Yeo KS, Begam T, Mohidin M *et al.* Identification of 5-methoxy-2-(diformylmethylidene)-3,3-dimethylindole as an anti-influenza a virus agent. *PLoS One* 2017; **12**. doi:10.1371/journal.pone.0170352.

2 Wu M, Pastor-Pareja JC, Xu T. Interaction between RasV12 and scribbled clones induces tumour growth

and invasion. *Nature* 2010; **463**: 545–548.

3 Cong X, Wang H, Liu Z, He C, An C, Zhao Z. Regulation of sleep by insulin-like peptide system in drosophila melanogaster. *Sleep* 2015; **38**: 1075-1083A.

4 Fedina TY, Arbuthnott D, Rundle HD, Promislow DEL, Pletcher SD. Tissue-specific insulin signaling mediates female sexual attractiveness. *PLoS Genet* 2017; **13**. doi:10.1371/journal.pgen.1006935.

5 Anding AL, Baehrecke EH. Vps15 is required for stress induced and developmentally triggered autophagy and salivary gland protein secretion in Drosophila. *Cell Death Differ* 2015; **22**: 457–464.
